# Analyzing Collaborative Governance Through Social Network Analysis: A Case Study of River Management Along the Waal River in The Netherlands

**DOI:** 10.1007/s00267-015-0606-x

**Published:** 2015-09-01

**Authors:** J. M. Fliervoet, G. W. Geerling, E. Mostert, A. J. M. Smits

**Affiliations:** Institute for Science, Innovation and Society, Faculty of Science, Radboud University Nijmegen, P.O. Box 9010, 6500 GL Nijmegen, The Netherlands; Deltares, P.O. Box 177, 2600 MH Delft, The Netherlands; Delft University of Technology, Stevinweg 1, 2628 CN Delft, The Netherlands

**Keywords:** Collaboration, Flood protection, Floodplain management, Nature restoration, River management, Social network analysis

## Abstract

Until recently, governmental organizations played a dominant and decisive role in natural resource management. However, an increasing number of studies indicate that this dominant role is developing towards a more facilitating role as equal partner to improve efficiency and create a leaner state. This approach is characterized by complex collaborative relationships between various actors and sectors on multiple levels. To understand this complexity in the field of environmental management, we conducted a social network analysis of floodplain management in the Dutch Rhine delta. We charted the current interorganizational relationships between 43 organizations involved in flood protection (blue network) and nature management (green network) and explored the consequences of abolishing the central actor in these networks. The discontinuation of this actor will decrease the connectedness of actors within the blue and green network and may therefore have a large impact on the exchange of ideas and decision-making processes. Furthermore, our research shows the dependence of non-governmental actors on the main governmental organizations. It seems that the Dutch governmental organizations still have a dominant and controlling role in floodplain management. This challenges the alleged shift from a dominant government towards collaborative governance and calls for detailed analysis of actual governance.

**Electronic supplementary material:**

The online version of this article (doi:10.1007/s00267-015-0606-x) contains supplementary material, which is available to authorized users.

## Introduction

A key challenge for environmental management is the number and diversity of the actors and sectors involved, each with their own perceptions, interests, and resources (Robinson et al. 2011). To address this challenge, multiple collaborative approaches have been developed, such as adaptive management (Folke et al. 2005; Pahl-Wostl et al. 2008; Stringer et al. 2006); multi-level governance (Blomquist 2009; Gruby and Basurto 2014; Lienert et al. 2013); community-based natural resource management (Kellert et al. 2000); network governance (Klijn et al. 1995; Rhodes 2007), and collaborative governance (Emerson et al. 2012). Despite their different foci, they share a number of characteristics:

1. They all address collaborations across organizational boundaries between diverse stakeholders, such as governmental actors, non-governmental actors, and/or citizens.
2. They all promise or expect better coordination between authorities and more integrated management (Emerson and Gerlak 2014).
3. They all assume a shift from state-centered, hierarchical top-down government towards less formalized governance by networks of interdependent stakeholders that extend beyond the government sector (“from government to governance”; Huitema and Meijerink 2014; Mostert 2015; Rhodes 1997; Termeer 2009).

Reasons given to collaborate include the limited resources of government: government simply does not have all the information, power, and finances necessary for environmental management, which makes it dependent on other stakeholders (Gray 1989; Huxham and Vangen 2005). Budget cuts over the past years have only increased this dependence. Moreover, involving diverse stakeholders can increase public support, reduce opposition, and improve implementation of government policy. And finally, there is the moral argument that involving stakeholders makes environmental management more democratic (Mostert et al. 2007; Stringer et al. 2006).

Empirical studies on the alleged shift from government to governance are scarce (e.g., Bodin and Crona 2009). In England, Watson et al. (2009) described how recent institutional reform in the water sector has actually strengthened control by state water agencies, despite the use of language emphasizing partnerships and collaborative governance. Non‐state actors and local authorities have been given substantial roles in the implementation of management measures, but the measures are still decided upon by national government and national government agencies, who also control implementation. Rather than increasing democracy and responsiveness, this has reduced public accountability because central government is able to deflect the blame when things go wrong (Watson et al. 2009).

The account given by Watson et al. (2009) raises a number of questions concerning the alleged shift from government to governance. The aim of the present article is to shed some more light on this issue and describe the complexity of the current collaborative and cross-boundary interactions between governmental and non-governmental actors concerning environmental management, using a case study approach. The method used is social network analysis (SNA: Borgatti et al. 2009). SNA analyzes social networks in terms of a set of nodes (e.g., individuals or organizations) and a set of ties between these nodes. It can provide insight in the position and role of individual actors in the network and help to identify central, coordinating, and bridging organizations whose activities connect actors that otherwise would not have been connected (Berkes 2009; Rathwell and Peterson 2012). The structure of ties between these actors gives insight in intra and inter-group collaboration (e.g., within government and between government and non-governmental actors) (Lienert et al. 2013). Finally, overall network properties, such as the number of ties compared to the number of possible ties, give insight in the potential for collaborative action and structural cohesion in the network (Olsson et al. 2004).

The case that will be analyzed is the maintenance of floodplains in the Dutch Rhine delta. The multi-functionality of these floodplains leads to interdependence of stakeholders with respect to the different functions, especially concerning flood protection and nature restoration (Fliervoet et al. 2013; Schindler et al. 2013). Both the “blue network” concerning flood protection and the “green network” concerning nature will be analyzed. The following questions will be addressed:

(i) Which actors are involved and what are their collaborative relationships to ensure flood protection (blue network) and/or reach nature objectives (green network)?
(ii) Which actors play a coordinating or bridging role?
(iii) What is the role of governmental versus non-governmental organizations in both networks?

The next section presents the case study and the methodology used. Subsequently, the results are presented. The article concludes with a discussion and conclusions on the main research questions.

## Method

### Case Study: Floodplain Management

**Fig. 1:**
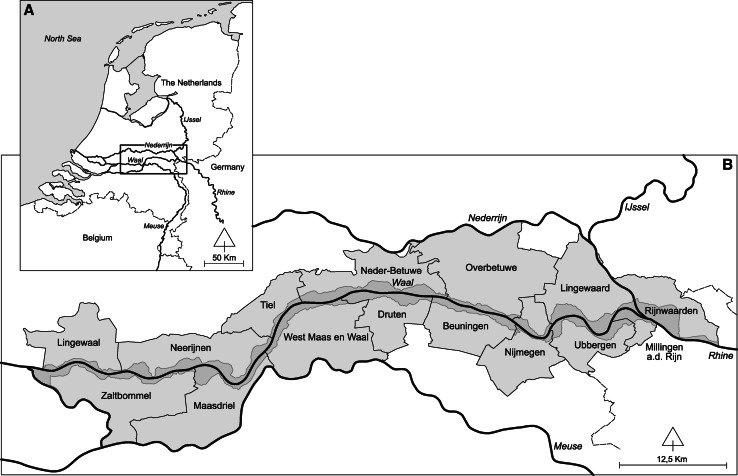
Study area (based on the Figure in Fliervoet et al. 2013): **a** location of the ‘WaalWeelde’ program in The Netherlands, **b** the specific locations of the fifteen municipalities (*light gray*) including the floodplain area (*dark gray*)

The case study that is central in this article is the maintenance of the floodplains of the River Waal, the main branch of the River Rhine in the Netherlands. The case study area includes one province and 15 municipalities and covers a river stretch of 80 km or 152 km^2^ (Fig. 1). The responsible authorities regarding flood protection are the State Water Agency (Directorate for Public Works and Water Management), which is responsible for the river itself and can regulate all activities in the floodplains that influence the water quality and quantity; and the Water Boards, which are responsible for the dikes and levees. Responsibility for maintaining and developing nature in the floodplains was decentralized in 2014 from the Ministry of Economic Affairs, which is also responsible for agriculture and nature policy, to the provincial governments. The provincial governments plan and implement EU Natura 2000 objectives based on the European legislation and allocate subsides for nature conservation. This may require changes in land use, which is regulated by the municipalities.

Alongside the authorities, a variety of private individuals, groups, and organizations have an interest in the maintenance activities in floodplains. These include nature conservation organizations; farmers; research institutes; and sand, gravel, and clay mining industries. The nature conservation organizations often deal with cultural heritage as well. Many farmers are also interested in maintaining biodiversity to be eligible for nature conservation subsidies. They are organized in farmers’ associations which combine agricultural activities with nature conservation.

In the 1990s, public and private stakeholders developed and implemented integrated plans to improve flood protection while restoring dynamic natural processes and safeguarding agriculture and recreational interests. These plans involved for instance the construction of new side channels through the floodplains that increased the discharge capacity of the river and offered space for nature. In this context, a program called ‘WaalWeelde’(in English: ‘Wealthy Waal’) was launched by the University of Nijmegen in 2006 and adopted by the provincial government in 2008 to connect public, private, and societal organizations in the planning and implementation phase of river management (Smits 2009). Based on a bottom-up approach, this integrated multi-player program aimed to develop a safer, more natural, and economically stronger riverine landscape.

Unfortunately, the integrated approach of the ‘WaalWeelde’ program has not been extended to the maintenance of the floodplains, which remains sectoral. This has resulted in new conflicts (Fliervoet et al. 2013). Increasingly, stakeholders recognized that floodplain management had become a very complex, dynamic, and fragmented issue and that more integrated and collaborative initiatives were needed to achieve sustainable floodplain management in the long-term (Fliervoet et al. 2013). This recognition led to the establishment of a taskforce ‘floodplain management’ in October 2011, which aimed to find an integrated, multi-player approach for the maintenance of the newly constructed multifunctional river landscapes, an approach that had been very successful in the early planning and implementation phases. In their final report, the taskforce proposed a new governance structure consisting of a ‘Waal Board,’ in which different governmental organizations would cooperate, and new private–private collaborations between land owners and nature conservation organizations, called ‘Stewardships.’ Meanwhile, the budgets of the governmental organizations declined, and in 2015, one national governmental organization was even abolished completely: the Government Service for Land and Water Management, which had 960 full time staff (2013 data: www.dienstlandelijkgebied.nl).

### Data Sources and Data Collection: Selection of Stakeholders

In this study, organizations were chosen as node level, as in Stein et al. (2011), Ingold (2011), and Knoke et al. (1996). The selection consisted of seventy organizations that already cooperated in the ‘WaalWeelde’ program, complemented with knowledge institutions and farmers associations (Fliervoet et al. 2013). The ‘WaalWeelde’ program included organizations based on their position, their role in decision process, and their reputation (cf. Knoke 1993). The key actors included governmental organizations, non-governmental organizations, businesses, knowledge institutions, and associations of farmers. Finally, the list was checked by the chairman of the ‘WaalWeelde’ Taskforce Floodplain Management.

The respondents were selected on the basis of the following criteria: (1) they represent one of the 70 listed organizations; (2) they have a high position in their organization, such as director or manager. With this selection, we ensured that respondents could represent the collaborative relations of their organization. Large organizations were split based on the level of departments or districts.

Respondents were asked by e-mail to fill in a survey about their collaborative relations. This e-mail was followed up by a reminder after 2 weeks and a phone call after 3 weeks. The survey consisted of an introduction stating the objective and questions on social characteristics, such as name and function of the respondent, name of the organization, scale of activities, and involvement or interest in flood protection (blue network) and/or nature (green network). Finally, respondents were asked to select from a list of 70 organizations, the organizations with which they interacted and to indicate the strength (frequency) of their interactions, for flood protection and nature objectives separately. The respondents could add missing organizations to the list. The options given for strength were (1) yearly or less, (2) quarterly, (3) monthly, and (4) on a weekly basis.

Of the 70 initial organizations, two did not exist anymore and four replied they were not involved in floodplain management. Of the remaining 64 organizations, 47 filled in the questionnaires, which constitutes a response rate of 73 percent. Seventeen organizations did not respond, including seven municipalities. Seven respondents added in total seventeen organizations. However, none of these organizations were added by more than one respondent. For this reason, we assume that the original list of organizations included the most relevant actors.

### Social Network Analysis

The survey data were modified before analysis in the software program UCINET (Borgatti et al. 2002). Three organizations were removed from the data because they indicated no involvement or collaborative interests in either flood protection or nature. Secondly, two respondents filled in the survey for the provincial government; therefore, one respondent was removed from the data. Ultimately, the data of 43 actors were analyzed regarding collaborative ties.

For the SNA in this paper, we used primarily reciprocated collaborative ties, meaning that both actors indicated that they collaborated. Since each tie depends on two actors, the data are more robust to reporting errors (Stein et al. 2011). In case actors indicated different meeting frequencies, the lowest frequency was used. The data were clustered by creating six groups based on the main organizational task or function (Ernoul and Wardell-Johnson 2013; Prell et al. 2008). These were (1) Flood protection (Fld) (*N* = 6); (2) Nature (Nat) (*N* = 11); (3) Agriculture (Agr) (*N* = 5); (4) Research institutes (Res) (*N* = 5); (5) Special interest groups (NGO/Businesses/Citizens) (Int) (*N* = 9); (6) Coordinators or spatial planning (Crd) (*N* = 7).

Table 1 shows the network metrics used in the results section. The networks were analyzed at three levels, i.e., (1) the network as a whole, (2) actor-groups, and (3) individual actors.

**Table 1:** Metrics used

Level	Metric	Definition	Interpretation and references
Whole-network properties	Density	Number of ties in the network divided by the maximum number ties possible (Borgatti et al. 2013)	The density metric analyzes the connectedness of the network, which is also known as network closure (Sandström and Rova 2010). The higher the network density, the more potential there is for collective action (Olsson et al. 2004). Bodin and Crona (2009) argue that less dense networks have clearly distinguishable subgroups, which could have negative effects on the capacity for collaborative processes among subgroups. However, a very high network density may decrease the groups’ effectiveness in collective action (Oh et al. 2004) because this can lead to homogenization of knowledge, which decreases the capacity for solving problems (Bodin and Norberg 2005)
Whole-network properties	Degree centralization	The general procedure involved in centralization is to look at the differences between the number of ties a node has (also known as degree centrality) of the most central point and those of all other points. Centralization, then, is the ratio of the actual sum of differences to the maximum possible sum of differences, also known as the approach of Freeman (1979) (Borgatti et al. 2013)	The degree centralization expresses how tightly the graph is organized around its most central point (Scott 1991) or, put differently, how ‘star-like’ the network structure is (Sandström and Rova 2010). A low degree centralization value indicates that many actors have spatially centralized positions in the network, which can refer to clearly distinguishable subgroups and a low level of network cohesion (Bodin and Crona 2009). A high degree of centralization indicates that one or a few actors (when the highest degree centrality is the same for more organizations) are central actors in a star-like configuration, see Fig. 1 from Gallemore and Munroe (2013)
Whole-network properties	Cross-boundary exchange	Number of ties connecting actors with different affiliations divided by the total number of connections in the network and expressed as percentage (Sandström and Rova 2010)	The cross-boundary exchange represents the ratio between collaborative ties within groups and between groups. It is a measure for the network heterogeneity. A low cross-boundary exchange indicates a relatively high tie density within groups (Sandström and Rova 2010)
Group properties	Group exchange	Reciprocal ties connected to one group divided by the total number of reciprocal ties in the network	This measure is used to identify dominant groups based on Ernoul and Wardell-Johnson (2013). The groups’ exchange (based on the group’s ties) within the whole network can be expressed in percentages
Group properties	Density by group (cross-table)	Density by group is the proportion of actual number of ties and the maximum possible number of ties within and between groups in a cross-table (Borgatti et al. 2013). The diagonal of the cross-table gives single group densities (supplementary material; Table SD-C and SD-D)	The higher the “Density by group”, the more potential for collective action between groups (Olsson et al. 2004). Density computed for all pairs of groups indicates mutual strong groups, as opposed to the group exchange, which defines the dominant groups of the total network
Actor properties	Degree (centrality)	Number of ties of an actor, often distinguishing between reciprocal ties, incoming ties (in-degree) and outgoing ties (out-degree) (Hanneman and Riddle 2005)	The number of ties an organization has (In-Degree, Out-Degree or reciprocal ties) has been shown to have a positive effect on that organization’s influence (Bodin and Crona 2009), but does not give information on the quality or frequency of the connection (Hanneman and Riddle 2005). A high number of Out-degree ties can indicate a high degree of dependence on other organization, a high number of In-degree ties can indicate a high degree of dependence by other organizations on the organization, and a high degree of reciprocal ties can indicate a high degree of interdependence
Actor properties	Betweenness (centrality)	Probability of an organization being on the shortest path between any two organizations in the network	The actor could act as a bridge between other actors who are not connected otherwise, which allows the actor to influence the information flows and act as a gatekeeper or mediator (Bodin and Crona 2009). These bridging organizations can play an important role in facilitating cross-scale interactions in environmental management (Rathwell and Peterson 2012)

## Results

This section presents the results of the social network analysis. First, we present and compare the whole-network properties of the blue and green networks. Subsequently, we focus on the involvement of the six groups of actors and define the most central players in Dutch floodplain management. Finally, we discuss the likely effect of the abolishment of the Government Service for Land and Water Management.

### Network Characteristics

**Fig. 2:**
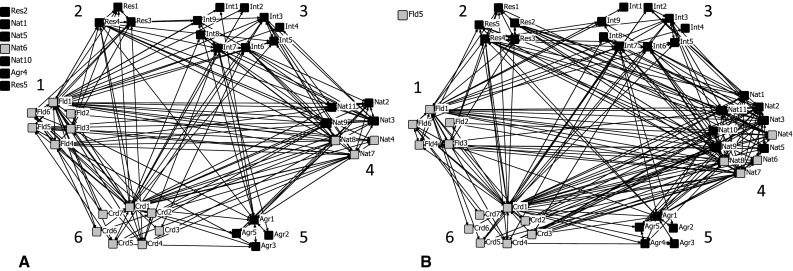
Social networks based on * all* reciprocal ties concerning flood protection objectives (**a**) and nature objectives (**b**). A *gray node* indicates a governmental organization and a *black node* a non-governmental organization. *Numbers* indicate the task or function of the six groups: *1* flood protection; *2* research institutes; *3* special interest groups; *4* nature; *5* agriculture; and *6* coordination or spatial planning

**Fig. 3:**
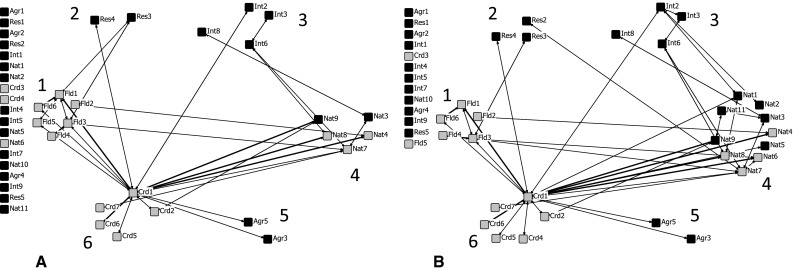
Social networks based on the *monthly* and *weekly* reciprocal ties concerning flood protection objectives (**a**) and nature objectives (**b**). *Bold lines* indicate the weekly ties. A *gray node* indicates a governmental organization and a *black node* a non-governmental organization. *Numbers* indicate the task or function of the six groups: *1* flood protection; *2* research institutes; *3* special interest groups; *4* nature; *5* agriculture; and *6* coordination or spatial planning

Table 2 presents the social network data describing the whole-network properties of the green and the blue networks, for all frequencies of collaboration and for monthly and weekly collaboration. The blue network for all frequencies consisted of 36 actors with reciprocal ties (out of 43 in total), and the green network of 42 actors (see also Figs. 2, 3). Even with the higher number of actors, the green network is denser by 30 % and has a higher degree of centralization. Both networks have a relatively high cross-boundary exchange and all groups are connected to the network, which altogether implies a heterogeneous network.

**Table 2:** Characteristics of the ‘blue’ (flood protection) and ‘green’ (nature) network based on the reciprocal ties and frequency of collaboration

	Size (number of nodes)	Density	Degree centralization	Cross-boundary exchange (%)	Total ties
Blue network (all frequencies)	36	0.175	0.516	75.32	316
Blue network (monthly and weekly)	24	0.033	0.340	70	60
Green network (all frequencies)	42	0.226	0.612	72.06	408
Green network (monthly and weekly)	30	0.044	0.403	65	80

The density and degree centralization values combined describe how well a network is connected. Both networks are well connected when focusing on all collaborative frequencies (Fig. 2). The shape of the networks tends to a centralized, wheel, or star-like network based on the high degree centralization scores (all tie frequencies). However, the higher degree centralization score of the green network suggests that this network is more centralized.

When focusing on the two highest tie-strength classes (monthly and weekly), both the blue and green whole-network indicators drop. This has a large impact on the connectedness of organizations in both networks, see for example the huge decrease of total ties. Additionally, twelve organizations drop out of the blue and green network on top of the already disconnected actors, especially actors of the research, agriculture, and special interest group (Fig. 3). Figure 3 also shows the large decrease of collaborative ties between the flood protection and nature group in both networks and the increase of the importance of Crd1, the Government Service for Land and Water Management. This actor holds the majority of the weekly reciprocal ties (thick lines in Fig. 3) and all remaining ties with the agricultural group. In both networks, the collaborative ties of the special interest group focus almost completely on organizations in the nature group. The organizations with a nature interest stay well connected in the green network, in spite of focusing on the more frequent collaborations, except actor Nat10 (Foundation ‘Lingewaard Natuurlijk’), which got disconnected.

### Specifications of the Groups’ Involvement

When we distinguish between governmental and non-governmental actors, we can clearly see the importance of the former (Table 3). While the number of government actors is smaller, they still account for 46 % (green network, all frequencies) to 75 % (blue network, monthly and weekly collaboration) of all ties. Flood protection and coordination of spatial planning are core government tasks, while nature is more a mixed governmental and non-governmental responsibility (Fig. 2). Government becomes even more important when low frequency ties are removed (Table 3; Fig. 3).

**Table 3:** The group exchange of the governmental and non-governmental organizations involved in the blue and green networks (in percentages)

Group number	Type of organization	Blue network (all frequencies)	Blue network (monthly and weekly)	Green network (all frequencies)	Green network (monthly and weekly)
1	Governmental organizations (*N* = 17)	54 (density = 0.382)	75 (density = 0.125)	46 (density = 0.346)	65 (density = 0.140)
2	Non-governmental organizations (*N* = 26)	46 (density = 0.123)	25 (density = 0.006)	54 (density = 0.197)	35 (density = 0.022)

Additionally, the density within the group is also indicated (see supplementary material for organizational attributes)

Almost all groups are well connected to each other when all tie strengths are included (Fig. 2). However, the flood protection and agricultural group show little collaboration between each other in either the blue or the green network. In addition, Fig. 2 shows the higher number of collaborative ties between the nature group on the one hand and the coordination and research group on the other in the green network as compared to the blue network.

Table 4 shows the actors grouped by their main tasks. In the blue network, the actors with interest in flood protection and nature have the highest degree of group exchange. Focusing on monthly and weekly ties only, the group exchange of actors responsible for coordination or spatial planning activities increases at the expense of research institutes and special interest groups. The green network shows a different pattern, with a high group exchange for the actors of the nature objective (36 %) and a lower group exchange for flood protection compared to the blue network. The actors involved in a coordinating role show a similar increase in group exchange when focusing on the stronger ties representing monthly and weekly collaborations, emphasizing their relative importance in the whole network.

**Table 4:** The group exchange in the blue and green networks (in percentages)

Group number	Main interest	Blue network (all frequencies)	Blue network (monthly and weekly)	Green network (all frequencies)	Green network (monthly and weekly)
1	Flood protection (*N* = 6)	22	27	13	16
2	Nature (*N* = 11)	24	22	36	35
3	Agriculture (*N* = 5)	7	3	8	2.5
4	Research (*N* = 5)	9	5	11	4
5	Special interest groups (*N* = 9)	18	10	15	12.5
6	Coordinators or spatial planning (*N* = 7)	20	33	17	30

The group density is higher within groups than between groups, especially in the green network (supplementary material; Table SD-C and SD-D). The coordinating group is an exception here, their highest tie density shifts along with the issue at stake (flood protection or nature), so the coordination group interacts most strongly with flood protection group in the blue network, and with the nature group in the green network. This applies also to the group of nature organizations, where the highest density scores are reached with the flood protection group in the blue network and with each other in the green network. Moreover, the group density scores show strong connections within the flood protection group and the low density scores among the organizations in the special interest group, which classifies this as weak connected group.

### The Central and Influential Organizations

The most central organizations in the blue and green network have been determined based on their number of reciprocal ties (degree centrality) and the amount of incoming ties (In-degree centrality) (supplementary material; Table SD-A and SD-B). The major difference between the two is that reciprocal degree shows mutual recognition while the In-degree values show the recognition of a collaborative actor by others only. In addition, the betweenness values for each actor are analyzed to identify bridging organizations.

The governmental actor Crd1 (*Government Service for Land and Water Management*) has the highest number of reciprocal and In-degree ties and the highest betweenness scores in both networks, except for the number of In-degree ties in the blue network, in which case it holds a third place (28 and 34 reciprocal ties in the blue and the green network respectively). Crd1 is the major broker among the coordinators of spatial planning and between this group and all other groups, especially the nature and flood protection group. Its central position and the bridging role are clearly visible in Fig. 3.

In the blue network, the second place, based on the number of reciprocal ties, is occupied by Fld 1, the *Delta Program*, with 27 reciprocal ties. This governmental actor was designated as the most important collaborative organization by the others (In-degree value). Fld1 is responsible for finding common ground for future flood protection measures to deal with climate change. So, collaboration between various actors is required, but also recognized by the others. In the green network, the second place is held by Nat7, the State Forestry Service, with 24 reciprocal ties. Also the betweenness value is relatively high, which expresses the influential role of the State Forestry Service (supplementary material; Table SD-B).

The actor Fld3, State Water Agency, is by mandate an important actor in river management with important management tasks and regulatory powers. It takes second place in terms of the number of In-degree ties in both networks, which shows that it is recognized by the other actors, but the ties are not reciprocal: the State Water Agency itself recognizes only a limited number of actors as collaborators. This suggest an unequal relation. Similarly, the Water Board (Fld6) appears in the top 5 for number of In-degree ties in both the blue and green network, but not for reciprocal ties. They also do not have an important bridging or coordinating function, according to their low betweenness scores.

Surprisingly, given its mandate, the actor Nat6, *province of Gelderland,* is not in the top 10 of reciprocal ties in both networks. It only scores relatively high with respect to In-degree ties in the green network, possibly because it holds some regulatory powers concerning nature protection.

### Discontinuation of the Most Central Actor

**Fig. 4:**
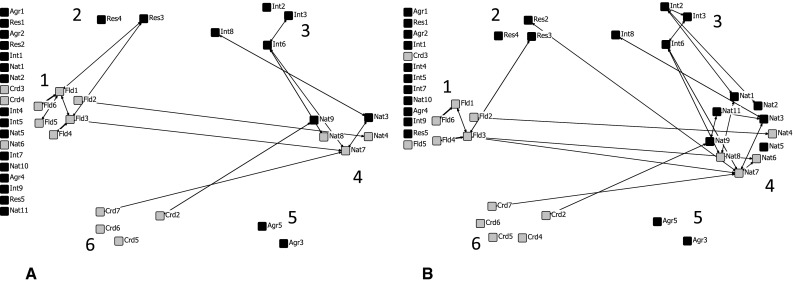
Social networks based on the *monthly* and *weekly* reciprocal ties concerning collaboration in the blue (**a**) and green network (**b**) after removing Crd1. *Bold lines* indicate the weekly ties. A *gray node* indicates a governmental organization and a *black node* a non-governmental organization. *Numbers* indicate the task or function of the six groups: *1* flood protection; *2* research institutes; *3* special interest groups; *4* nature; *5* agriculture; and *6* coordination or spatial planning

Due to state budget cuts, the Government Service for Land and Water Management (Crd1) has been abolished on 1 March 2015. The effects of removing this governmental actor can be seen by comparing Fig. 3, which shows the situation until 1 March 2015 (reciprocal ties, weekly or monthly), with Fig. 4, in which we have removed Crd1. Assuming everything else remaining the same, all farmers’ associations and many other organizations will become isolated and in fact drop out of the networks. In total, six organizations will drop out of the blue network and seven out of the green network. The bridging function of Crd1 between the flood protection and nature group will be lost. Especially the blue network will become very fragmented; the green network will still be held together by the group with a nature affiliation.

## Discussion

### Complexity of Collaborative Floodplain Management

In the article, two networks for maintaining floodplains were analyzed, one with a focus on flood protection and the other on nature, in order to increase insight in the complexity of natural resources management and the alleged changed role of government. The analysis has shown, first, that both the blue and green networks are well-connected and heterogeneous networks, with complex structural patterns. Cross-boundary exchange (75 and 72 %, respectively), network density, and degree centralization are relatively high compared to other studies (Sandström and Rova 2010; Stein et al. 2011).

The relatively high network density improves the potential for collective action and collaboration (Olsson et al. 2004) because well-connected networks facilitate communication, foster mutual trust, and help to prevent or manage conflicts (Bodin et al. 2006). Sandström and Carlsson (2008) showed that high tie density promotes joint-action, especially when many ties exist between different types of actors (e.g., between recreational fishermen and governmental officials). The green network has a higher network density than the blue network, indicating a greater potential for collective action on nature issues. The high ratio of relationships between different types of actors can be observed in both the blue and the green network, even for monthly and weekly ties.

The high density of the networks may also have some adverse effects. An actor with too many ties may feel obligated to please all or most of its collaborative partners. This may reduce the actor’s possibilities for action (Bodin and Crona 2009) and slow down progress, resulting in ‘partnership fatigue’ (Huxham et al. 2000, p. 347). Moreover, a tangle of collaborative ties may reduce transparency and accountability (Huxham et al. 2000). In addition, a dense network implies relatively few contacts with outsiders that may have different points of views. This may reduce the capability to innovate (Bodin and Norberg 2005).

### The Dense Green Network and the Role of Nature Organizations

The size of the green and the blue network indicates that mutual recognition of collaborative partners is stronger in the former than in the blue network (42 vs. 36, Table 2, based on reciprocal ties). The organizations not included in the blue network were mostly organizations with a main interest in nature objectives or research institutes with an ecological interest.

The clustering in groups is supported by the high group density scores within the groups. In the blue network, three groups play an equally dominant role, flood protection, nature and coordination actors, especially when we focus on weekly and monthly ties. The green network is, perhaps not surprisingly, mostly dominated by the group of nature organizations, which collaborate a lot with the coordinating group and research institutes.

Overall, the respondents believed more strongly in the added values of collaborative partnerships for nature objectives than for flood protection objectives, reflected in the higher green network density. There are several explanations for this:

- The management of the flood protection objectives could be seen as a governmental issue, while interest for nature conservation and restoration issues is more spread and recognized by non-governmental organizations.
- Nature organizations need (strong) partnerships to reach their objectives (Warner and van Buuren 2009), whereas water agencies have strong regulatory powers and their own funding.
- Nature organizations have much more experience with collaborative processes than water agencies (Koontz and Thomas 2006).

### Central Actors

Analysis of the most intensive collaborative ties identified the weak relationship between the nature and flood protection organizations as shown by the few weekly and monthly ties between the two groups (Fig. 3). To our knowledge, this has not yet been formally analyzed for floodplain management, although the fragmented governance of Dutch floodplains is “general knowledge” (Fliervoet et al. 2013; Wiering and Van de Bilt 2006).

In both networks, the most central organization based on degree centrality is the Government Service for Land and Water Management (Cdr1), a national governmental organization established for coordination, collaboration, and implementation of spatial planning, i.e., a bridging organization (Berkes 2009). The central position of Cdr1 in both networks makes the organization a perfect candidate to facilitate the idea of public–public collaboration (Waal Board, see paragraph 2.1). Unfortunately, in the beginning of 2015, this organization was disbanded due to national state budget cuts. The second most influential role is designated to the Delta Program (Fld1) in the blue and the State Forestry Service (Nat7) in the green network. These organizations have much influence on the current collaborative network and could act as bridges between other actors who are not connected otherwise, given their betweenness value (Bodin and Crona 2009).

It is remarkable that the main authorities for nature were not recognized as important collaborative partners (reciprocal ties): they were not even in the top-10. The provincial government (Nat6) only recognized a couple of collaborative partners within the green network. In addition, their recognition by other actors (In-degree ties) is also relatively low for a main authority. Their low ranking is probably caused by the recent decentralization of the nature policies from the ministry of Economic Affairs to the provincial governments in 2014, which was maybe not yet fully recognized by all actors. In contrast, the well-established State Water Agency (region East) (Fld3) was recognized as an important collaborative partner by many others (top-2 position based on In-degree ties), but did not reciprocate this recognition. This low ratio of in- versus out-degree ties shows the power and independence of the State Water Agency and also the provincial government: they do not need the other organizations to implement their policies and select only a small number of collaborative partners. To a lesser extent, this also applies to the position of the Water Boards.

### The Consequences of Removing a Central, Governmental Actor

The states’ discontinuation of the most central governmental organization (Cdr1) will most likely have a large impact on the current collaborative structures, especially on the flood protection network. Assuming all else remaining the same, both structural integration and inclusiveness (Lockwood et al. 2010) will decline. Farmers’ associations and spatial planning agencies (municipalities) will become disconnected. The number of links between different groups, especially the nature and flood protection group, will decrease. This may not only reduce opportunities for collective action, but also make floodplain maintenance less integrated (Lockwood et al. 2010). According to Lauber et al. (2008), it may reduce the exchange of ideas, decrease the access to funding, and reduce the influence of certain stakeholders. Exchange of ideas through the whole network is hampered by less network cohesion, whereas in particular the municipalities and associations of farmers will be disempowered by the loss of the bridging function of Cdr1. Crd1 no longer brings together diverse goals which will constrain the funding opportunities, especially funding for nature, which depends on third parties as it is often coupled with other goals.

### Implications for the Government’s Role

Our data indicate that different groups of interest are connected, but it also supports the idea that governmental organizations still control and occupy central positions in the network, like in United Kingdom (Watson et al. 2009, see introduction). This challenges the alleged shift from (hierarchical) government to (collaborative) governance. Yes, there is a lot of collaboration, but there is also still a lot of hierarchical government. The question is whether this is necessarily bad and whether it could be different. Government can play different roles in collaborations. Government bodies can be an active participant and use its regulatory powers to implement its own policy and reach its own objectives; it can coordinate and facilitate, like Crd1 did; and it can stimulate collaboration hierarchically, for instance, by changing the rules, selectively empowering collaborators with fewer resources, and threatening to impose regulation if no results are achieved (cf. the “shadow of hierarchy”: e.g., Börzel and Risse 2010; Héritier and Lehmkuhl 2008). If government takes on the first role and tries to run the show on its own, it could frustrate collaboration, but if it takes on the second or third role, or both, it could potentially stimulate collaboration. In any case, government still is important and most likely will remain so.

Because of the importance of government, attempts to improve the maintenance of the Dutch floodplains should involve the governmental organizations. Watson et al. (2009) argue that there is a greater need to recognize the integration of land and water management at the local scale and to develop appropriate institutional arrangements for both policy making and policy implementation. In our case, both the green and the blue networks rely on similar collaborative relationships. This offers opportunities for integrating the maintenance of flood protection and nature objectives at the local level and to collaboratively develop an appropriate policy for sustainable floodplain management. The basic idea is that a collaborative forum of governmental organizations at higher levels can support on-the-ground efforts of local groups (Margerum 2007).

The discontinuation of Cdr1 creates an opportunity to simplify and restructure the network to ultimately achieve a better integration of flood protection and nature management in floodplains. At the local or regional level, a coordinating or facilitating role could be played by the State Forestry Service (region East), mainly based on their central position in both networks. However, a coordinating or facilitating role demands for an actor with a wide and a more or less neutral perspective on the maintenance issues. These requirements seems to fit better with the tasks and function of the provincial government rather than the State Forestry Service (region East), as well to keep the distance between European and national policies and local actors as small as possible.

Another candidate to take on a coordinating role would be the Delta Program (Fld1), which holds the second and third most central position in, respectively, the blue and green network. The Delta Program started in 2009 as a collaborative program involving public and private organizations, but it is now responsible for a yearly, returning program to improve the flood protection levels and ensure fresh water supply in the context of climate change. Despite their main focus on water and planning, they have the capacity to develop an integrated, long-term maintenance vision for the floodplains. These ideas should be studied more in-depth to prove the feasibility. In the end, there would be one collaborative network concerning floodplain management.

Still, it is worth emphasizing that effective collaborative governance requires that governmental organizations do not become too dominant and recognize others as collaborative partners. This is an important factor, alongside the need for sharing responsibilities and knowledge, flexibility, building trust and setting up learning environments for collaborative governance (Emerson and Gerlak 2014). In other words, collaborative governance cannot be achieved without a change of thinking and acting of the central government and its executive agencies (Watson et al. 2009).

## Conclusions

In sum, this study demonstrated the complexity of collaborative relationships based on a case study on the maintenance of the Dutch floodplains, using a social network approach. The complexity was explored by focusing on the networks regarding two conflicting issues: flood protection and nature. The organizations in both networks are well connected and diverse in terms of goals, whereby the nature organizations possess the most collaborative ties. The dense green (nature oriented) network includes more organizations and collaborative relationships than the blue (flood protection) network. This indicates that the potential for collective action is higher in the green network. Analysis of the most frequent relationships (monthly and weekly ties) showed that few frequent collaborative ties existed between flood protection and nature groups.

The most central organization in both networks was Crd1, a governmental organization focused on coordinating land and water management. This organization had links with many different interest groups and played an important bridging role between the nature and flood protection oriented organizations. Quite remarkably, this organization has been abolished early 2015 due to state budget cuts in a time period where collaboration is framed as a solution. Removing a central actor from a dense network will have consequences, especially in this case. Assuming all else remaining the same, the structural integration of both networks will decrease, especially the bridging function of Crd1 between the water agencies and nature organizations will be lost. Additionally, groups, such as the associations of farmers and municipalities, will become disconnected, which may decrease their participation in and influence on decision making. On the positive side, the discontinuation of coordinating governmental actors will give opportunities to simplify and restructure the complex collaborative network, for example, through a more facilitating role of the provincial government, who could support on-the-ground efforts of local groups.

In both the blue and green networks, governmental actors have the highest number of reciprocal ties and dominate the collaboration. The powerful and independent role of the main authorities can be deduced from the differences between the number of incoming and outgoing ties, reflecting recognition *by* others and *of* others respectively. Therefore, we argue that currently there is no shift from ‘government to governance’ with respect to the maintenance of the Dutch floodplains. To achieve more collaborative governance, new collaborative relationships have to be developed, which requires time, effort, and recognition of non-governmental actors as full partners.

## Electronic supplementary material

Supplementary material 1 (DOCX 58 kb)
