# Supplementary material for: Analyzing Collaborative Governance Through Social Network Analysis: A Case Study of River Management Along the Waal River in The Netherlands
Source: Environ Manage. 2015 Sep 1;57:355–67. doi: 10.1007/s00267-015-0606-x (PMC4712244; doi:10.1007/s00267-015-0606-x)
Supplement: Supplementary file 1 — Supplementary material 1 (DOCX 58 kb) [file 267_2015_606_MOESM1_ESM.docx]

**Supplementary Data to paper:**

**Analyzing collaborative governance through social network analysis: a case study of river management along the Waal River in The Netherlands**

**Author names and affiliations**

J.M. Fliervoet^a^*, G.W. Geerling^ab^, E. Mostert^c^, A.J.M. Smits^a^

^a^ Institute for Science, Innovation and Society, Faculty of Science, Radboud University Nijmegen, P.O. Box 9010, 6500 GL Nijmegen, The Netherlands

^b^ Deltares, P.O. box 177, 2600 MH Delft, The Netherlands

^c^ Delft University of Technology, Stevinweg 1, 2628 CN Delft, The Netherlands

*Corresponding author. Postal address: Institute for Science, Innovation, and Society, Faculty of Science, Radboud University Nijmegen, P.O. Box 9010, 6500 GL Nijmegen, The Netherlands; Tel.: +31 24 365 26 84; E-mail address: j.fliervoet@fnwi.ru.nl

Table of Contents

[1. Centrality measures and organizational attributes 2](#_Toc419895804)

[*1.1 Blue network* 2](#_Toc419895805)

[*1.2 Green network* 3](#_Toc419895806)

[2. Analysis of density values for groups 3](#_Toc419895807)

[*2.1 Density by group in the blue network* 3](#_Toc419895808)

[*2.2 Density by group in the green network* 3](#_Toc419895809)

1. Centrality measures and organizational attributes

*1.1 Blue network*

Table SD-A shows the results of the network analysis regarding **flood protection** objectives. The table shows two different degree centralities: (1) the out- and in-degree and (2) the degree centrality based on reciprocal ties in the network. The table is sorted to the degree reciprocal scores. Additionally, the betweenness values are analyzed for the reciprocal ties. The organizational attributes indicate the abbreviation and type of organization; Gov. = governmental organization or nongov. = non-governmental organization. The abbreviation relates to the defined task or function of the group; flood protection (Fld); nature (Nat); agriculture (Agr); research institutes (Res); special interest groups (NGO / Businesses / Citizens) (Int); coordinators or spatial planning (Crd).

Table SD-A: Degree centrality measures and organizational attributes regarding flood protection objectives.

| **nr** | **Abbreviation** | **Stakeholder** | **OutDeg** | **Indeg** | **Degree - reciprocal** | **Between - Reciprocal** | **gov/nongov** |
| --- | --- | --- | --- | --- | --- | --- | --- |
| 9 | Crd1 | Government Service for Land and Water Management | 40 | 29 | 28 | 132 | gov. |
| 7 | Fld1 | Delta Program: Rivers | 33 | 30 | 27 | 120 | gov. |
| 31 | Nat7 | State Forestry Service: region east | 41 | 17 | 17 | 27 | gov. |
| 30 | Int7 | Citizen platform: *Spiegelgroep WaalWeelde* | 42 | 16 | 16 | 53 | Nongov. |
| 27 | Res4 | Radboud University of Nijmegen | 21 | 20 | 15 | 19 | Nongov. |
| 32 | Nat8 | State Forestry Service: region east: district river landscape | 36 | 16 | 15 | 21 | gov. |
| 13 | Nat3 | Federation for nature and environment of Gelderland | 29 | 19 | 14 | 25 | Nongov. |
| 1 | Agr1 | Association of agriculture and nature management: *‘Lingestreek’* | 37 | 12 | 12 | 29 | Nongov. |
| 42 | Fld6 | Water Board: *‘Rivierenland’* | 20 | 20 | 11 | 27 | gov. |
| 10 | Int3 | Federation of sand, gravel, clay and limestone mining industries | 27 | 12 | 10 | 4 | Nongov. |
| 12 | Nat2 | Foundation of Gelderse landscapes and castles | 28 | 12 | 10 | 7 | Nongov. |
| 22 | Int6 | K3Delta | 30 | 13 | 10 | 19 | Nongov. |
| 28 | Fld3 | Directorate for Public Works and Water Management: East | 12 | 30 | 10 | 6 | gov. |
| 33 | Nat9 | ARK Foundation | 17 | 19 | 10 | 6 | Nongov. |
| 41 | Fld5 | Water Board: *‘Rijn en IJsel’* | 20 | 14 | 10 | 8 | gov. |
| 16 | Crd4 | Municipality of Nijmegen | 20 | 15 | 9 | 4 | gov. |
| 29 | Fld4 | Directorate for Public Works and Water Management: East: district south | 23 | 14 | 9 | 4 | gov. |
| 14 | Crd2 | Municipality of Beuningen | 14 | 14 | 8 | 3 | gov. |
| 8 | Res3 | Deltares | 10 | 16 | 7 | 2 | Nongov. |
| 17 | Crd5 | Municipality of Rijnwaarden | 11 | 15 | 7 | 4 | gov. |
| 21 | Int5 | Citizen platform (Hoogwaterplatform) | 17 | 11 | 7 | 2 | Nongov. |
| 23 | Nat4 | Ministry of Economic Affairs | 13 | 18 | 6 | 1 | gov. |
| 2 | Res1 | Alterra: research institute | 5 | 17 | 5 | 0 | Nongov. |
| 39 | Int9 | Citizen platform: *‘de Verrekijkers’* | 8 | 13 | 5 | 0 | Nongov. |
| 43 | Nat11 | World Wildlife Fund | 8 | 14 | 5 | 1 | Nongov. |
| 18 | Crd6 | Municipality of Tiel | 10 | 11 | 4 | 0 | gov. |
| 19 | Crd7 | Municipality of Zaltbommel | 5 | 12 | 4 | 0 | gov. |
| 35 | Int8 | Foundation Symbiose | 16 | 7 | 4 | 0 | Nongov. |
| 36 | Agr3 | Association *‘Rijnstrangen’* | 5 | 8 | 4 | 1 | Nongov. |
| 38 | Agr5 | Association of agriculture and nature management: *‘de Capreton’* | 19 | 8 | 4 | 0 | Nongov. |
| 24 | Fld2 | Ministry of Infrastructure and the Environment | 4 | 22 | 3 | 0 | gov. |
| 3 | Agr2 | Association of three farmers: *"Opheusden en Omgeving"* | 2 | 4 | 2 | 0 | Nongov. |
| 5 | Int1 | Cascade, association of sand and gravel industries | 4 | 14 | 2 | 0 | Nongov. |
| 6 | Int2 | Dekker groep | 5 | 18 | 2 | 0 | Nongov. |
| 15 | Crd3 | Municipality of Lingewaard | 5 | 14 | 2 | 0 | gov. |
| 20 | Int4 | Federation for anglers | 3 | 13 | 2 | 0 | Nongov. |
| 4 | Res2 | Federation for forest and nature (O+bn) | 0 | 9 | 0 | 0 | Nongov. |
| 11 | Nat1 | FREE (Foundation for Restoring European Ecosystems) Nature | 0 | 13 | 0 | 0 | Nongov. |
| 25 | Nat5 | Natuurmonumenten | 0 | 17 | 0 | 0 | Nongov. |
| 26 | Nat6 | Province of Gelderland: program nature | 0 | 19 | 0 | 0 | gov. |
| 34 | Nat10 | Foundation *‘Lingewaard Natuurlijk’* | 0 | 6 | 0 | 0 | Nongov. |
| 37 | Agr4 | Association of agriculture and nature management: ‘*Rijk Maas en Waal’* | 0 | 6 | 0 | 0 | Nongov. |
| 40 | Res5 | Wageningen University & Research Centre | 0 | 13 | 0 | 0 | Nongov. |

*1.2 Green network*

Table SD-B shows the results of the network analysis regarding **nature** objectives. The table shows two different degree centralities: (1) the out- and in-degree and (2) the degree centrality based on reciprocal ties in the network. The table is sorted to the degree reciprocal scores. Additionally, the betweenness values are analyzed for the reciprocal ties. The organizational attributes indicate the abbreviation and type of organization; Gov. = governmental organization or nongov. = non-governmental organization. The abbreviations relate to the defined task or function of the group; flood protection (Fld); nature (Nat); agriculture (Agr); research institutes (Res); special interest groups (NGO / Businesses / Citizens) (Int); coordinators or spatial planning (Crd).

Table SD-B: Centrality measures and organizational attributes regarding nature objectives.

| **No.** | **Abbreviation** | **Stakeholder** | **OutDeg** | **Indeg** | **Deg. Reciprocal** | **Betweenness - reciprocal** | **gov/nongov** |
| --- | --- | --- | --- | --- | --- | --- | --- |
| 9 | Crd1 | Government Service for Land and Water Management | 40 | 35 | 34 | 204 | gov. |
| 31 | Nat7 | State Forestry Service: region east | 40 | 25 | 24 | 89 | gov. |
| 7 | Fld1 | Delta Program: Rivers | 33 | 27 | 23 | 94 | gov. |
| 32 | Nat8 | State Forestry Service: region east: district river landscape | 37 | 21 | 19 | 34 | gov. |
| 13 | Nat3 | Federation for nature and environment of Gelderland | 31 | 20 | 17 | 30 | Nongov. |
| 27 | Res4 | Radboud University of Nijmegen | 20 | 24 | 17 | 37 | Nongov. |
| 30 | Int7 | Citizen platform: *Spiegelgroep WaalWeelde* | 42 | 16 | 16 | 76 | Nongov. |
| 1 | Agr1 | Association of agriculture and nature management: *‘Lingestreek’* | 38 | 15 | 15 | 39 | Nongov. |
| 12 | Nat2 | Foundation of Gelderse landscapes and castles | 28 | 17 | 15 | 18 | Nongov. |
| 33 | Nat9 | ARK Foundation | 25 | 20 | 15 | 14 | Nongov. |
| 22 | Int6 | K3Delta | 30 | 16 | 12 | 11 | Nongov. |
| 10 | Int3 | Federation of sand, gravel, clay and limestone mining industries | 27 | 13 | 11 | 7 | Nongov. |
| 23 | Nat4 | Ministry of Economic Affairs | 13 | 22 | 11 | 9 | gov. |
| 43 | Nat11 | World Wildlife Fund | 16 | 14 | 11 | 4 | Nongov. |
| 4 | Res2 | Federation for forest and nature (O+bn) | 20 | 14 | 10 | 10 | Nongov. |
| 11 | Nat1 | FREE (Foundation for Restoring European Ecosystems) Nature | 14 | 17 | 10 | 6 | Nongov. |
| 28 | Fld3 | Directorate for Public Works and Water Management: East | 12 | 29 | 10 | 9 | gov. |
| 29 | Fld4 | Directorate for Public Works and Water Management: East: district south | 24 | 16 | 10 | 5 | gov. |
| 16 | Crd4 | Municipality of Nijmegen | 17 | 17 | 9 | 3 | gov. |
| 42 | Fld6 | Water Board: Rivierenland | 19 | 24 | 9 | 17 | gov. |
| 14 | Crd2 | Municipality of Beuningen | 16 | 15 | 8 | 2 | gov. |
| 15 | Crd3 | Municipality of Lingewaard | 11 | 15 | 8 | 1 | gov. |
| 25 | Nat5 | Natuurmonumenten | 12 | 20 | 8 | 1 | Nongov. |
| 26 | Nat6 | Province of Gelderland: program nature | 12 | 21 | 8 | 2 | gov. |
| 38 | Agr5 | Association of agriculture and nature management: *‘de Capreton’* | 21 | 13 | 8 | 3 | Nongov. |
| 2 | Res1 | Alterra: research institute | 7 | 20 | 7 | 3 | Nongov. |
| 17 | Crd5 | Municipality of Rijnwaarden | 11 | 16 | 7 | 6 | gov. |
| 34 | Nat10 | Foundation *‘Lingewaard Natuurlijk’* | 10 | 9 | 7 | 1 | Nongov. |
| 6 | Int2 | Dekker groep | 12 | 19 | 6 | 1 | Nongov. |
| 8 | Res3 | Deltares | 9 | 18 | 6 | 3 | Nongov. |
| 40 | Res5 | Wageningen University & Research Centre | 8 | 17 | 6 | 4 | Nongov. |
| 21 | Int5 | Citizen platform (Hoogwaterplatform) | 12 | 10 | 5 | 1 | Nongov. |
| 35 | Int8 | Foundation Symbiose | 15 | 6 | 4 | 0 | Nongov. |
| 37 | Agr4 | Association of agriculture and nature management: ‘*Rijk Maas en Waal’* | 8 | 8 | 4 | 0 | Nongov. |
| 39 | Int9 | Citizen platform: *‘de Verrekijkers’* | 7 | 12 | 4 | 0 | Nongov. |
| 24 | Fld2 | Ministry of Infrastructure and the Environment | 3 | 20 | 3 | 0 | gov. |
| 36 | Agr3 | Association *‘Rijnstrangen’* | 7 | 9 | 3 | 0 | Nongov. |
| 3 | Agr2 | Association of three farmers: *"Opheusden en Omgeving"* | 2 | 4 | 2 | 0 | Nongov. |
| 18 | Crd6 | Municipality of Tiel | 3 | 11 | 2 | 0 | gov. |
| 19 | Crd7 | Municipality of Zaltbommel | 3 | 12 | 2 | 0 | gov. |
| 5 | Int1 | Cascade, association of sand and gravel industries | 2 | 15 | 1 | 0 | Nongov. |
| 20 | Int4 | Federation for anglers | 5 | 13 | 1 | 0 | Nongov. |
| 41 | Fld5 | Water Board: Rijn en IJssel | 0 | 17 | 0 | 0 | gov. |

2. Analysis of density values for groups

*2.1 Density by group in the blue network*

Table SD-C: the cross-table indicates the density values between and among groups within the blue network (all frequencies). The highest density score of each group (rows) is emphasized (bold) indicating the strongest crosslink with another group (or itself).

| Group number | 1 | 2 | 3 | 4 | 5 | 6 |
| --- | --- | --- | --- | --- | --- | --- |
| 1. Flood protection (N= 6) | **0.600** | 0.258 | 0.067 | 0.267 | 0.148 | 0.405 |
| 1. Nature (N= 11) | **0.258** | 0.164 | 0.091 | 0.073 | 0.182 | 0.195 |
| 1. Agriculture (N= 5) | 0.067 | 0.091 | **0.300** | 0.040 | 0.089 | 0.114 |
| 1. Research (N= 5) | 0.267 | 0.073 | 0.040 | **0.300** | 0.089 | 0.114 |
| 1. Interest groups (N= 9) | 0.148 | 0.182 | 0.089 | 0.089 | **0.222** | 0.127 |
| 1. Coordination or spatial planning (N= 7) | **0.405** | 0.195 | 0.114 | 0.114 | 0.127 | 0.333 |

*2.2 Density by group in the green network*

Table SD-D: the cross- table indicates the density values between and among groups within the green network (all frequencies). The highest density score of each group (rows) is emphasized (bold) indicating the stronges crosslink with another group (or itself).

| Group number | 1 | 2 | 3 | 4 | 5 | 6 |
| --- | --- | --- | --- | --- | --- | --- |
| 1. Flood protection (N= 6) | **0.400** | 0.258 | 0.033 | 0.233 | 0.111 | 0.286 |
| 1. Nature (N= 11) | 0.258 | **0.473** | 0.200 | 0.291 | 0.212 | 0.364 |
| 1. Agriculture (N= 5) | 0.033 | 0.200 | **0.500** | 0.080 | 0.044 | 0.171 |
| 1. Research (N= 5) | 0.233 | 0.291 | 0.080 | **0.600** | 0.089 | 0.143 |
| 1. Interest groups (N= 9) | 0.111 | 0.212 | 0.044 | 0.089 | **0.250** | 0.143 |
| 1. Coordination or spatial planning (N= 7) | 0.286 | **0.364** | 0.171 | 0.143 | 0.143 | 0.238 |
